# Antibiotic Prophylaxis in the Management of Vesicoureteral Reflux

**DOI:** 10.1155/2008/825475

**Published:** 2008-09-29

**Authors:** Marc Cendron

**Affiliations:** Department of Pediatric Urology, Children's Hospital, Harvard School of Medicine, 300 Longwood Avenue, Boston, MA 02115, USA

## Abstract

Antibiotic prophylaxis has been, since 1960s, one of the management options in treating vesicoureteral reflux. The purpose of this review article is to provide a concise overview of the rational for antiobiotic prophylaxis and to discuss the various agents used. Some of the current controversies regarding use of antibiotics for reflux will also be presented.

## 1. INTRODUCTION

Once vesicoureteral reflux (VUR) has been diagnosed, the basic premise in management is to prevent further ascending urinary tract infections (UTI) which may, if left untreated, lead to pyelonephritis. Pyelonephritis, in turn, would lead to potential renal damage [1]. Based on the work of Jean Smellie et al. in 1960s, use of antibiotic prophylaxis was found to be helpful in reducing the recurrence rate of urinary tract infection in children with VUR [2]. Subsequently, several long-term studies have demonstrated the validity of the concept [3–7]. The basis for the antibiotic prophylaxis in patients with VUR was the fact that, ultimately, reflux in low grades (I through III) was recognized to resolve over time and thus maintenance on low-dose antibiotic would prevent or reduce the risk of urinary tract infection until such time that the reflux would disappear [8]. The goal of this article is, therefore, to review the management of VUR using antibiotic prophylaxis, its advantages and disadvantages based on a review of the literature. The various antibiotic used for prophylaxis in VUR will be discussed.

## 2. THE EVOLUTION OF ANTIBIOTIC PROPHYLAXIS IN THE MANAGEMENT OF PATIENTS WITH VUR

Regurgitation of urine from the bladder up into the ureter and renal collecting system has been recognized since early times [9]. VUR became identified as an etiologic factor for pyelonephritis from the classic studies carried out by Hutch, who in 1952, studied a group of paraplegic patients diagnosed with neurogenic dysfunction of the bladder and VUR. Reflux of infected urine into the upper urinary tract was postulated to be the cause of chronic and progressive renal damage [10]. Later, in 1959, Hodson observed that reflux seemed to be more common in children with urinary tract infections and that there was a correlation between reflux and chronic pyelonephritis as documented by VCUG (voiding cystourethrogram) and IVU (intravenous urogram) [11]. As the association between VUR and urinary tract infection became more established, additional experimental studies demonstrated the role of bacterial infection in causing renal damage in patients found to have VUR [12–14].

Historically, the initial approach to treating patients with reflux was observational without continuous antibiotic. Treatment was offered only as infections occurred. Unfortunately this approach demonstrated that renal damage could occur in patients who had had only one infection and that further renal damage was more likely to occur in kidneys that were noted to have parenchymal lesions but could also occur in normal kidneys. Lenaghan reported that most infections occurred within the first five years after the initial diagnosis [15]. In light of the high rate of new kidney damage noted in children treated with intermittent antibiotic therapy, it was suggested that prophylactic antibiotic be used. Lenaghan's conclusions were confirmed by the international reflux study in children which also demonstrated a high rate of new scar formation in children who were observed off continuous antibiotic prophylaxis but with known reflux [16]. This study found that new renal damage occurred in 12.5% of children with normal kidneys, whereas 62% of scarred kidneys showed progression of the damage as infections were treated. A number of subsequent studies showed that progression of scarring in patients with reflux could occur in the face of recurrent urinary tract infection [17, 18]. N. P. Goldraich and I. H. Goldraich, in 1992, showed that, in a large prospective study children with VUR of grades I through V treated with antibiotic prophylaxis, a relatively low rate of new scar formation (3%) was found and this was seen only in cases where urinary tract infection occurred [19]. The Southwest Pediatric Nephrology Study Group demonstrated that in a relatively small group of patients with grade 1–3 reflux followed for five years, 12 patients (10.7%) developed new scars on Intravenous Urogram intravenous urogram (IVU) in the face of breakthrough infections [20]. Skoog et al. observed a large cohort of patients (545) on continuous low-dose antibiotic prophylactic for up to ten years. A relatively low rate of progressive scarring in the kidneys was noted (0.5%) mostly occurring in children with breakthrough infection [21]. Current recommendations for antibiotic prophylaxis in children have been formalized by the AUA guideline panel on the management of primary VUR in children [22]. Recommendations from the guidelines were that children with VUR grade I through IV could be initially managed medically with continuous antibiotic prophylaxis because of fewer risks, in the short term, and that surgery would be recommended for children who experienced breakthrough infections.

More recently the concept of stopping antibiotic prophylaxis after a certain age has been evaluated as parents have increasingly become weary of long-term medication intake and concerns have been raised about side effects and bacterial resistance. Based on the findings that, by age 4, renal scarring was unlikely to occur in the face of urinary tract infection, cessation of antibiotic prophylaxis was felt to be reasonable in children beyond the age of 5 [23, 24]. Cooper et al. evaluated a group of 51 patients with reflux with a mean age of 8.6 years who were not treated with prophylactic antibiotic [25]. Despite the fact that reflux persisted in the majority of these patients, only a small number of patients (11%) developed a subsequent urinary tract infection. No new renal scars were noted as documented by ultrasound, which, however, may not be the most accurate modality to ascertain for renal lesions. Unfortunately, no long-term double blinded randomized study has been carried out to compare the efficacy of antibiotic prophylaxis versus no antibiotic prophylaxis in patients diagnosed with VUR based on the degree of reflux. In addition, the data is still not entirely clear with regard to the comparison between surgical therapy and antibiotic prophylaxis. The International Reflux Study in Children (IRSC) failed to demonstrate a clear advantage of any of these two forms of management [26]. The major limitations of the study were that not all grades of reflux were managed by either modality, since higher grades of VUR were treated surgically thus introducing a serious selection bias. Currently, medical management of VUR still remains commonly practiced for younger patients with lower grades of VUR as the randomized studies are being set up.

## 3. ANTIBIOTICS USED FOR PROPHYLACTIC TREATMENT IN PATIENTS WITH VUR

A relatively small number of antimicrobials are used to treat urologic conditions in children, the most common ones being used for antibiotic prophylaxis in the face of VUR are trimethoprim-sulphamethoxasole (TMP/SMX), nitrofurantoin, and penicillin derivatives amoxicillin. The advantage of these antibiotics is that their active form or metabolites are excreted in the urine thus keeping the urine free of bacteria. Guidelines for administrations will not be reviewed but it should be kept in mind that only penicillin derivatives are used in younger children under 2 months of age because the immaturity of the newborn liver and kidneys results in a slower metabolism and excretion of these medications [27]. Allergic reactions to antimicrobials should always be a concern. A family history is helpful in determining which child may actually be allergic to a medication. Allergic reactions manifest themselves as either urticaria, diffuse skin rash, or, more rarely, anaphylaxis. Subcutaneous skin testing may resolve the question of an allergic reaction to medication but since the testing itself may be associated with some risk of allergic reaction this should be performed under controlled conditions by an allergist. A 5% cross allergenicity between penicillin and cephalosporin should also be recognized [28]. In general, however, children who have mild or delayed allergic reaction to one of these classes of antimicrobials are usually able to tolerate agents in other classes.

Bacterial drug resistance is a growing problem worldwide. In 1980s, widespread recognition of the issue came about with the widely reported vancomycin resistant staphylococcal aureus infection found in cases of community acquired infection [29]. The incidence of drug resistance has clearly increased over the last 20 years and has become a major health issue. Control of antimicrobial resistance is clearly a multifaceted task involving hospital policy, individual provider practice, and patient compliance. Guidelines that have been developed by the Joint Committee on Antimicrobial Resistance to help decrease the emergence of drug resistance organism include a careful use of broad spectrum antibiotic, the tailoring of therapy to sensitivity profiles, and the avoidance of unnecessary prolonged therapy [30].

We will review the specific antimicrobials used in patients with VUR for prophylaxis of infections. These include penicillins, TMP-SMX, and nitrofurantoin.

The penicillin class of antimicrobials includes natural penicillins (V and K), amino penicillins (ampicillin and amoxicillin), the beta-lactamase resistant penicillins (methicilin, nafcilin, oxacillin, dicloxacillin), and the antipseudomonal penicillins (carbenicillin, ticarcillin, azlocilin, and mezlocillin). The natural penicillins are used to prevent and treat infections caused by group A streptococci and S pneumonia. These medications are now rarely used for prophylaxis because of their limited commercial availability and because resistance patterns have increased. Amino penicillins have become the most commonly used penicillins. They are the drug of choice in treating enterococcal urinary tract infection and can be used for prophylaxis in infants under age 2 months. These agents are usually effective against most bacteria susceptible to Penicillin G as well as some Penicillin G resistant gram negative bacilli [31]. Amino penicillins are excreted primarily by the kidney. Ampicillin is available both in oral and intravenous formulation but Amoxicillin is only available as an oral agent. Amoxicillin has better bioavailability than ampicillin because more of it is absorbed from the digestive tract. A higher percentage of unabsorbed oral ampicillin remaining in the gut alters gut flora frequently leading to GI upset and diarrhea which may be a concern in younger children. A higher rate yeast infections has also been noted. Beta-lactamase-resistant penicillins are usually not used for prophylaxis as are the antipseudomonal penicillins. Reactions to penicillins are relatively rare and include hypersensitive reactions, neurotoxicity, nephrotoxicity, and hematologic toxicity [32].

Trimethoprim/sulfamethoxazole is a combination agent that inhibits the production of bacterial folic acid, thereby blocking DNA synthesis. It is the most widely used outpatient antibiotic agent used for prophylaxis in children with vesicoureteral reflux. While trimethoprim (Primsol) alone has a similar antibacterial activity to sulfamethoxazole, the spectrum of activity expands when the drugs are combined. In addition, resistance develops less quickly in the combined formulation than with either drug alone [33]. Sulfamethoxazole and trimethoprim are both absorbed rapidly after all administration. The majority of sulfamethoxazole undergoes hepatic metabolism to inactive metabolites, while approximately half of the absorbed trimethoprim is converted hepatically into inactive metabolites. Most of the active and inactive drug is then excreted by the kidney into the urine [34].

The adverse reactions associated with this combination of drug are most often caused by the sulfa component. These reactions include hypersensitivity reaction (ranging from a mild rash to severe Stevens-Johnson exfoliative reaction which may be severe and life threatening), severe photosensitivity reaction, and hematologic toxicity that presents as agranulocytosis or hemolytic anemia, prompting the recommendation that a complete blood count be obtained in children who are taking sulfa medications for extended periods of time. Sulfonamides are contraindicated in children younger than 2 months of age because the sulfa moiety from the drug can displace bilirubin from its natural albumin binding site, predisposing infants to hyperbilirubinemia [35].

Nitrofurantoin is an agent widely used for the prophylactic management of VUR. While it is most commonly administered in an oral formulation, a parenteral form is also available. It is well absorbed orally and undergoes significant hepatic degradation to inactive metabolites. Because of its extended metabolism and relatively poor tissue penetration, nitrofurantoin is used solely as a urinary tract desinfectant since it will achieve bacteriocidal concentration only in urine. [33]. Although its exact mechanism of action is unknown, nitrofurantoin is thought to inhibit bacterial acetyl coenzyme A, thereby interfering with carbohydrate metabolism. It may also disrupt bacterial cell wall synthesis. Nitrofurantoin is usually effective in treating staphylococci, streptococci and most community acquired gram negative uropathogens. Despite its widespread use, bacteria rarely develop resistance to nitrofurantoin. This is most probably due to the fact that the drug does not achieve significant levels in intestinal or vaginal tissues and does not alter the normal flora in these areas [36]. The most common side effect associated with nitrofurantoin is GI upset with nausea, vomiting, or diarrhea. Use of the microcrystalline formula and administration with meals usually eliminates these side effects. A rare, more severe side effect is pulmonary fibrosis, which is most likely to occur after long-term therapy (months to years) and can present acutely with episodic coughing and/or dyspnea which would warrant a full evaluation. Hemolytic anemia can occur in patients with glucose-six-phosphate dehydrogenase deficiency as well as in infants under one month of age.

Cephalosporins are rarely used for antibiotic prophylaxis unless patients have shown resistance to TMP/SMX. Because of their broad activity against community acquired pathogen, this class of antimicrobial is usually used for surgical prophylaxis or for acute treatment of urinary tract infection. Cephalosporins provide a reasonable antimicrobial activity against most gram positive and gram negative bacteria. Use of prophylaxis is usually not recommended as the medications tend to be expensive. However, some cephalosporins including Cephalexin have prolonged urinary concentration and may be helpful for short-term antibiotic prophylaxis.

Fluoroquinolones inhibit action of the essential bacterial enzyme DNA gyrase which consequently prohibits maintenance of the superhelical twist in the double stranded DNA causing rapid cell death [37]. One important clinical aspect of the antibacterial spectrum of fluoroquinolones is their effectiveness in treating hospital acquired organisms. These antimicrobials have good pharmacokinetic qualities which include rapid absorption from the intestinal tract, good tissue penetration, and good intracellular diffusion. Long-term use of fluoroquinolones in a pediatric population has been reported to be effective and safe in patients with cystic fibrosis [38]. Ciprofloxacin in children seems to be well tolerated with no significant evidence of arthropathy, bone abnormalities, and no serious adverse side effects [39]. However, fluoroquinolones have not become an acceptable form of antibiotic prophylaxis given their expense and given the risk for possible emergence of resistant organism to this class of antimicrobials [40].

## 4. THE DOWN SIDES OF ANTIBIOTIC PROPHYLAXIS IN THE MANAGEMENT OF VUR

Three interrelated issues come into play when considering prophylactic antibiotic therapy for VUR: compliance, efficacy, and long-term side-effects of chronic antibiotic administration. Let us examine each of these issues. A recent report indicate that long-term administration of a daily antibiotic may not be carried out as carefully and consistently as one would hope. Hensle et al. in a review of patterns of care based on health insurance data showed that only 17% of patients were at least 80% compliant with prophylactic treatment [41]. In addition, as time goes by, compliance with antibiotic intake has been shown to go down by the first year follow-up visit [42]. Efficacy of antibiotic therapy in reducing the rate of urinary tract infection is hard to evaluate as no long-term, randomized placebo-controlled studies have, to date, been published. The Cochran Database Systematic Review meta-analysis reported that there was no significant difference in risk for urinary tract infection between daily antibiotic prophylaxis and no prophylaxis or between intermittent (3 days per week) prophylaxis and no prophylaxis [43]. The report also found no difference in the risk of renal parenchymal damage between the various treatment options. In addition the review indicated that 30 to 50% of patients on antibiotic prophylaxis were reported to have a UTI within 5 years. In a recent multicenter, randomized study of antibiotic prophylaxis treatment of patients with lower grades of VUR, Garin et al. showed a similar one year urinary infection rate between patients who were treated with or without antibiotic prophylaxis: 23.6% of children with grade 1 through 3 reflux received antibiotics and acquired a urinary tract infection while 22.4% of those on no antibiotic prophylaxis acquired one. Interestingly, those patients on antibiotic prophylaxis were found to have a higher rate of pyelonephritis upon follow-up [44]. Recurrent infections are, therefore, a worrisome issue in patients with VUR maintained on antibiotic prophylaxis. Sjöström reported a rate of breakthrough urinary tract infections in patients with reflux up to 47% of case [45]. Whether or not these infections are harmful in the long term is unclear but this persistent ability to acquire urinary tract infection clearly brings into question the efficacy of antibiotic prophylaxis and the role of host susceptibility to infections. If one looks at the ability of prophylactic antibiotic to reduce the risk for renal scarring, Reddy et al. reported no difference in occurrence of renal damage amongst patients with VUR randomized to receive either antibiotic prophylaxis or no antibiotics [46]. Finally, it would appear that outcomes seem to be rather similar in patients randomly assigned to medical or surgical management [47]. In a recent open-label, randomized study from Italy, antibiotic prophylaxis was not found to be effective in reducing the rate of pelonephritis recurrence and the incidence of renal damage in young children with VUR grades II, III, IV [48].

## 5. CONCLUSION

Antibiotic prophylaxis still seems to be a reasonable management option after initial diagnosis of VUR especially in children under age five who may be more susceptible to renal damage if an ascending urinary tract infection occurs. Issues of noncompliance, questionable efficacy, potential side effects and allergic reactions, and antimicrobial resistance have now brought into question use of antibiotic prophylaxis in the management of VUR. Further, uncertainty is built into the fact that prediction of reflux resolution varies from patient to patient and may involve other factors than anatomic ones. The complex nature of the interaction between VUR and UTIs and their effects on the kidneys make the identification of those patients at risk for ascending urinary tract infection and subsequent renal damage the biggest challenge in managing VUR. Since the available data is still not sufficient in providing objective guidelines for use of antibiotic prophylaxis in managing VUR, further long-term, randomized placebo-controlled studies are clearly needed to allow better insight into this form of management. In the first year after diagnosis, consistent, low-dose administration of antibiotics may be helpful in reducing the rate of urinary tract infection provided that the right antibiotic is administered keeping in mind the caveat of such a treatment, mainly the rising rate of antimicrobial resistance [48]. It should be noted that cranberry juice may have a role in reducing the rate of UTIs [49]. Parents of children diagnosed with VUR should be apprised of the potential side effects of the medications used for prophylaxis of UTIs and of the other options available for treatment. Reassurance as to the relatively low rate of complication rate seen with antibiotic prophylaxis should be emphasized. Until such studies show unequivocally that antibiotic prophylaxis is ineffective in preventing urinary tract infection and renal damage, antibiotic prophylaxis still remains a viable option in the management of VUR [50].
